# Impact of the COVID-19 pandemic on self-harm and self-harm/suicide ideation: population-wide data linkage study and time series analysis

**DOI:** 10.1192/bjp.2023.76

**Published:** 2023-11

**Authors:** Euan Neil Paterson, Lisa Kent, Dermot O'Reilly, Denise O'Hagan, Siobhan M. O'Neill, Aideen Maguire

**Affiliations:** Centre for Public Health, Queen's University Belfast, Belfast, Northern Ireland; Administrative Data Research Centre Northern Ireland (ADRC-NI), Queen's University, Belfast, Northern Ireland; Public Health Agency, Belfast, Northern Ireland; School of Psychology, Ulster University, Coleraine, Northern Ireland

**Keywords:** COVID-19, self-harm, self-harm/suicide ideation, administrative data, mental health

## Abstract

**Background:**

The COVID-19 pandemic and associated lockdowns were predicted to have a major impact on suicidal behaviour, including self-harm. However, current studies have produced contradictory findings with limited trend data.

**Aims:**

Nine years of linked individual-level administrative data were utilised to examine changes in hospital-presenting self-harm and ideation (thoughts of self-harm or suicide) before and during the pandemic.

**Method:**

National self-harm registry data were linked to demographic and socioeconomic indicators from healthcare registration records (*n* = 1 899 437). Monthly presentations of self-harm or ideation were split (pre-COVID-19 restrictions: April 2012 to February 2020; and during restrictions: March to September 2020). Auto-regressive integrated moving average (ARIMA) models were trained in R taking into consideration trends and seasonal effects. Forecast (‘expected’) monthly values were compared with ‘actual’ values, stratified by demographic factors and method of harm.

**Results:**

The number of individuals presenting with self-harm or ideation dropped significantly at the beginning of the pandemic (March–May 2020), before returning mostly to expected trends from June 2020. Stratified analysis showed similar presentation trends across most demographic subgroups except for those aged over 65 years, living alone or in affluent areas, where presentations remained unaffected, and those aged under 16 years, where numbers presenting with self-harm or ideation increased above expected levels.

**Conclusions:**

Although population trends show an overall drop in presentations before a return to ‘normal’ from June 2020, the demographic profile of those presenting with self-harm or ideation varied significantly, with increases in children under the age of 16 years. This highlights important potential target groups who may have been most negatively affected by the pandemic.

The public health impact of, and response to, the emergence of COVID-19 in 2019 have few parallels globally in terms of scope or scale, with the closest perhaps being measures taken in response to the influenza pandemic of 1918–1919.^1^ Public health measures in the UK included physical distancing, social and physical isolation, the wearing of oral-nasal facemasks, limitations on face-to-face business practices (including front-line health and social care), restrictions on travel, and infection surveillance and tracking measures, all with the intention of reducing the spread of infection and the likelihood of exceeding healthcare capacity.^2^ The aggregate effect of these pandemic-response measures constitutes a population-level exposure of an unprecedented type and magnitude.

This exposure to response measures and to the COVID-19 infection itself are expected to have a negative impact on population mental health, including risk of self-harm. Isolation, loss, fear of infection and anxiety have increased with the emergence of COVID-19 and these are all risk factors for self-harm.^3^ However, the evidence to date is conflicting. An international living systematic review on the impact of the pandemic on self-harm and suicidal behaviour has found no evidence of an increase in global self-harm and evidence of a fall in hospital-presenting suicidal behaviour.^4^ A UK-based study found a decrease in rates of hospital presentations for self-harm in the first 6 weeks of the pandemic, before rates returned to normal.^5^ But this study was limited to a small regional analysis in one area of England and examined numbers of presentations rather than numbers of individuals presenting. A UK-wide household panel survey running since the first ‘lockdown’ in March 2020 has found no change in self-reported thoughts of self-harm or suicide during the pandemic and an e-cohort study examining both primary care and secondary care contacts for self-harm during the pandemic in Wales found a decrease in all contacts.^6,7^ Experts have recommended that longer-term, population-wide monitoring of self-harm behaviour during the pandemic is required.^8^

Northern Ireland is unique in that it contains the only national population-wide registry of self-harm and self-harm/suicide ideation in the world. This study will utilise these data to examine the impact of the COVID-19 pandemic and associated response on hospital-presenting self-harm and self-harm/suicide ideation to determine (a) whether the COVID-19 pandemic and associated restrictions have had an effect on total number of individuals presenting with self-harm or self-harm/suicide ideation and (b) whether the presentation profile for self-harm or self-harm/suicide ideation changed during the first 7 months of the pandemic; these 7 months included the UK-wide ‘stay at home’ order (March–May 2020) advising people to work and school from home and restrict travel (which became known as the ‘UK lockdown’), the new ‘stay alert’ order (from May 2020), the introduction of ‘support bubbles’ allowing lone adults to mix with one other household (from June 2020) and restriction of face-to-face teaching (March–September 2020).

## Method

### Study design and participants

This population-based data-linkage study extracted demographic data on the entire Northern Ireland population registered on the national central health card registration system (National Health Application and Infrastructure Service (NHAIS)) and linked this to the Public Health Agency's Northern Ireland Registry of Self-Harm (NIRSH) within the Northern Ireland Trusted Research Environment (NI TRE) at the Business Services Organisation's Honest Broker Service. Exact data linkage was performed using the Trusted Third Party methodology within the NI TRE and data were de-identified and pseudonymised prior to release to the research team. Participant consent was not required for this study.

The NHAIS contains information on all patients registered with a primary care physician in Northern Ireland, and as Northern Ireland has a universal, tax-financed, free-at-the-point-of-service healthcare system almost the entire population are registered on the NHAIS. This data-set includes basic demographic details and address information for each patient as well as their health and care number (HCN), a unique identifier that facilitates exact matching to other health system data-sets. Sex was defined as male/female as registered with the NHAIS. Age was defined at presentation and grouped into five age bands (<16 years, 16–24, 25–44, 45–64, 65+ years). The NHAIS database was also used to identify people living alone (single-person households) as this is a known risk factor for poor mental health.^9^ The patient's address in March 2020 was used to categorise their area of residence into tertiles of deprivation based on the income domain of the Northern Ireland Index of Multiple Deprivation (tertile 1: most deprived; tertile 3: least deprived) and into ‘urban’ (Northern Ireland's two largest cities, Belfast and Derry), ‘intermediate’ (all other towns/villages) and ‘rural’ (population <1000 and open countryside) areas based on an official classification of settlements.

The NIRSH contains information on all presentations to every hospital emergency department in Northern Ireland for self-harm or self-harm/suicide ideation (referred to throughout as ‘ideation’) from April 2012 to September 2020.^10^

Within the NIRSH, ‘self-harm’ is derived from the World Health Organization/Euro Multicentre Study Working Group's definition of ‘parasuicide’, which includes any ‘act with non-fatal outcome in which an individual deliberately initiates a non-habitual behaviour, that without intervention from others will cause self-harm, or deliberately ingests a substance in excess of the prescribed or generally recognised therapeutic dosage, and which is aimed at realising changes that the person desires via the actual or expected physical consequences’.^10^ The NIRSH excludes accidental self-harm and acts of self-harm by those with intellectual disabilities because level of intent is difficult to ascertain, but uniquely includes acts of ideation, which are presentations by people who have experienced thoughts of self-harm or suicide but have not acted on them. The data are extracted from emergency department records by trained data collectors using standardised criteria. Data captured include HCN, method, any ideation and admission/discharge details. The main method of self-harm at presentation was defined as: cutting, overdose, hanging/other (which included more severe methods of harm such as attempted drowning or jumping from a high place) and ideation only (which included individuals who had experienced thoughts of self-harm and/or suicide, where no physical act of harm had taken place). Data were divided into pre-COVID-19 restrictions (April 2012 to February 2020) and during the first 7 months of the restrictions/UK lockdown (March to September 2020).

### Statistical analysis

Data pre-processing was performed in STATA SE15. Statistical analyses were performed using R4.2.1 for windows. Packages included tidyverse and forecast.^11,12^ Total monthly counts were created for number of presentations of, and number of individuals presenting with, self-harm or ideation. Counts were also stratified by sex, age, living alone, area-level deprivation and rurality, method of self-harm and type of presentation.

#### Pre-existing trends: April 2012 to February 2020

Line graphs were generated to visualise change in monthly counts over time for number of presentations and number of individuals, stratified by sex, age, method of self-harm and type of presentation. Individuals with missing demographic or socioeconomic data were included in the analysis with a ‘missing’ category included for each variable as required.

#### Impact of restrictions on hospital-presenting self-harm/ideation

In the absence of widely accepted guidelines for the reporting of time series analyses, this paper adheres to the recommendations set out by Jandoc et al^13^ and utilised in previous COVID-19 trend analysis.^14^ Data were separated into pre-pandemic/restrictions (April 2012 to February 2020) and during pandemic/restrictions (March 2020 to September 2020). The ARIMA model was trained on monthly frequency data using the auto.arima() function (detailed code available at GitHub: https://github.com). The algorithm was permitted to iteratively attempt to fit on differenced data (to remove trend) and first seasonal difference (to remove seasonal trend) and automatically choose the best fit. A transfer function (i.e. shape of the impact after initial introduction of intervention) was not added to the ARIMA model as restrictions over the time period studied were subject to change. The trained ARIMA models were then used to forecast number of individuals presenting with self-harm or ideation in the ‘during restrictions’ timeframe. The expected (i.e. forecast) monthly values and upper and lower 80% and 95% confidence limits were extracted and plotted against actual monthly values. This allowed identification of actual monthly values that lie outside of the confidence limits for the expected values. Observed-to-expected ratios (and 95% confidence intervals) were also calculated.

### Ethical approval

The project was approved by the Honest Broker Service Governance Board and received ethical approval from Yorkshire & The Humber – Sheffield Research Ethics Committee (20/YH/0254). The authors assert that all procedures contributing to this work comply with the ethical standards of the relevant national and institutional committees on human experimentation and with the Helsinki Declaration of 1975, as revised in 2008. Patient consent was not required for this study, which used anonymised administrative data.

## Results

A total of 41 400 individuals had 111 779 presentations to an emergency department for self-harm or ideation in Northern Ireland from April 2012 to September 2020, with 21 567 males (60 285 presentations) and 19 833 females (51 494 presentations) represented in the study data. The study population as a whole had a mean of 2.70 presentations (male: 2.74; female: 2.56). The majority of presentations were for self-harm (*n* = 74 791, 66.9%), with approximately half as many for ideation (*n* = 36 988, 33.1%).

A summary of the demographic and socioeconomic characteristics for the cohort is presented in Table 1. Individuals who presented with either self-harm or ideation tended to be male (52.1% male), middle aged (25–44 years, 37.1%), not live alone (84.0%) and live in deprived areas (11.6% from the least deprived area) or urban areas (only 15.6% from rural areas). Differences in characteristics of those who presented with self-harm or ideation were examined. Self-harm identified anyone who ever presented with self-harm (*n* = 32 108) and ideation anyone who ever presented with ideation (*n* = 17 245) over the study period. These groups are not mutually exclusive. A higher proportion of those in the ideation group were male (61.7%), with all other demographic indicators similar. The most common method of self-harm was overdose (54.1%), with approximately a fifth of all individuals presenting with ideation only (22.4%).

**Table 1:** Sociodemographic characteristics and number (%) of individuals with self-harm or ideation (thoughts of self-harm or suicide) at any time between April 2012 and September 2020 at first presentation

Characteristic	Category	Any self-harm or ideation	Any self-harm	Any ideation
		*n* = 41 400 (100%)	*n* = 32 108 (100%)	*n* = 17 245 (100%)
Sex^a^	Female	19 833 (47.9)	16 460 (51.3)	6613 (38.4)
	Male	21 567 (52.1)	15 648 (48.7)	10 632 (61.7)
Age group, years^b^	10–16	2742 (6.6)	2218 (6.9)	761 (4.4)
	16–24	12 026 (29.1)	9796 (30.5)	4556 (26.4)
	25–44	15 348 (37.1)	11 838 (36.9)	6828 (39.6)
	45–64	9897 (23.9)	7342 (22.9)	4455 (25.8)
	65+	1387 (3.4)	914 (2.9)	645 (3.7)
Household occupancy	Single person	6313 (15.3)	4721 (14.7)	3357 (19.5)
	>1 person	34 779 (84.0)	27 165 (84.6)	13 727 (79.6)
	missing	308 (0.7)	222 (0.7)	161 (0.9)
Urbanicity	Urban	11 610 (28.0)	9116 (28.4)	4929 (28.6)
	Intermediate	22 776 (55.0)	17 772 (55.4)	9404 (54.5)
	Rural	6450 (15.6)	4783 (14.9)	2651 (15.4)
	missing	564 (1.4)	437 (1.4)	261 (1.5)
Area-level deprivation	(most deprived) 1	12 045 (29.1)	9419 (29.3)	5224 (30.3)
	2	23 889 (57.7)	18 492 (57.6)	9821 (57.0)
	(least deprived) 3	4780 (11.6)	3678 (11.5)	1874 (10.9)
	missing	686 (1.7)	519 (1.6)	326 (1.9)
Self-harm method	Overdose	22 376 (54.1)	22 376 (69.7)	n/a
	Cutting only	5871 (14.2)	5871 (18.3)	n/a
	Hanging/Other	3861 (9.3)	3861 (12.0)	n/a
	Ideation only	9292 (22.4)	n/a	n/a

n/a: not applicable.

a. Male and female *n* and % are calculated based on the gender reported at the first presentation within the data-set. A number of individuals had male and female sex reported at different presentations (*n* = 694, 1.68%).

b. Participants <10 years of age are not included in the analysis as the outcome is rare in this group.

### Trends: April 2012 to September 2020

**Fig. 1:**
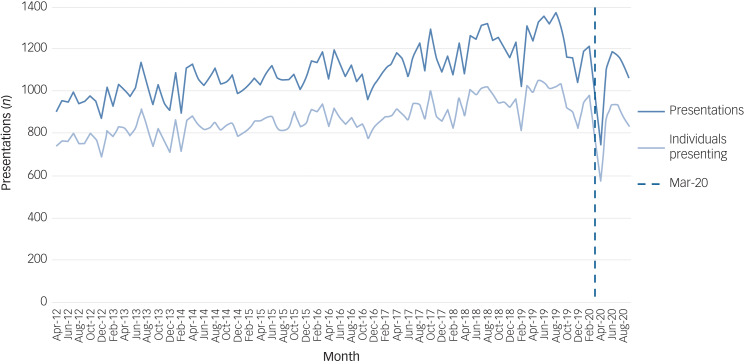
Number of presentations and number of individuals presenting with self-harm or ideation (thoughts of self-harm or suicide) between 2012 and 2020 (the dashed vertical line depicts the onset of the pandemic in March 2020).

Figure 1 shows the number of individuals (and count of number of presentations) presenting to an emergency department with self-harm or ideation each month from April 2012 to September 2020. There is a general upward trend in both total presentations and numbers of individuals presenting over the pre-pandemic period. There is a sharp decrease in number of presentations and number of individuals presenting in March 2020 (initiation of first UK COVID-19-related lockdown) before trends appear to return to normal from May 2020. The rate of presentations and number of individuals presenting per 100 000 population during the same time period are shown in Supplementary Fig. 1, available at https://doi.org/10.1192/bjp.2023.76. Rates per 100 000 population followed the same trend observed in both total presentations and numbers of individuals presenting. In 2019 approximately 56.45 individuals per 100 000 presented each month to an emergency department with self-harm or ideation (full data in Supplementary Table 1). Rates were lowest in April 2020, at 33.97 per 100 000 individuals, but had returned to expected levels of approximately 55.50 per 100 000 individuals in June 2020.

Within the stratified groups (Supplementary Figs 2–7), the trends generally followed the pattern of the whole population above, with some exceptions in the very old and very young (Supplementary Fig. 3), those living alone (Supplementary Fig. 4), those in affluent areas (Supplementary Fig. 5) and those with more severe methods (Supplementary Fig. 7), where the trends appeared unaffected by the March 2020 lockdown.

### Change in self-harm and ideation presentations during pandemic/restrictions

**Fig. 2:**
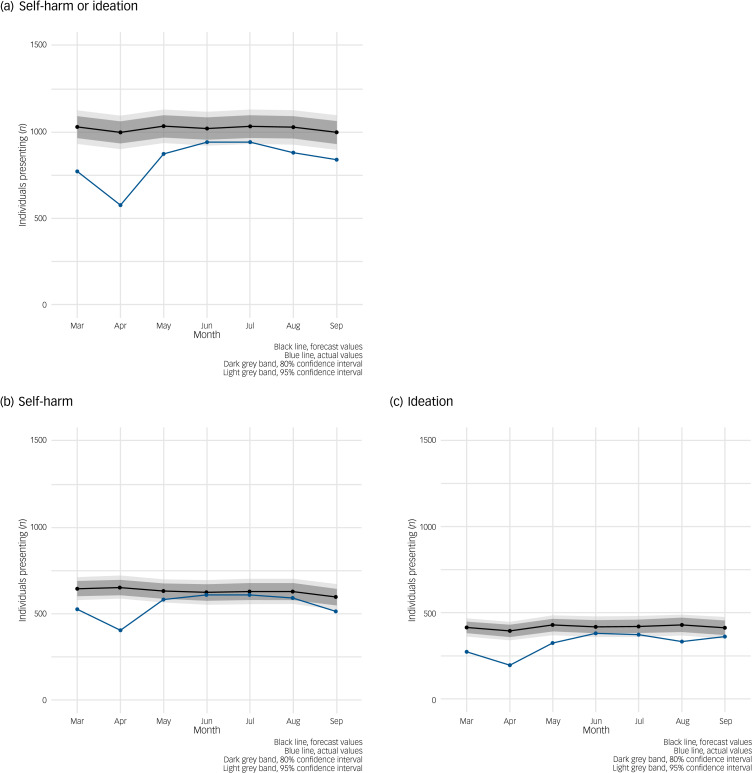
Auto-regressive integrated moving average (ARIMA) illustrating forecast versus actual numbers of individuals presenting with self-harm or ideation (thoughts of self-harm or suicide) during the first seven months of COVID-19 pandemic/restrictions in Northern Ireland. (a) Individuals presenting with self-harm or ideation. (b) Individuals presenting with self-harm. (c) Individuals presenting with ideation.

ARIMA models examining trends in the number of individuals presenting with self-harm or ideation show that, at a population level, there appears to be a lower-than-expected number of individuals presenting to an emergency department in the two months immediately after the initiation of restrictions in Northern Ireland (March–April 2020) before numbers approach expected levels in May to July but fall slightly below predicted levels in August and September (Fig. 2). In five out of the seven months studied, the number of individuals presenting with self-harm or ideation was below the forecast values. When stratified, the number of individuals presenting with self-harm was as expected in four out of the seven follow-up months (Fig. 2(b)), whereas the number of individuals presenting with ideation was as expected in only two out of the seven follow-up months (Fig. 2(c)), all lower than expected in all other months.

**Fig. 3:**
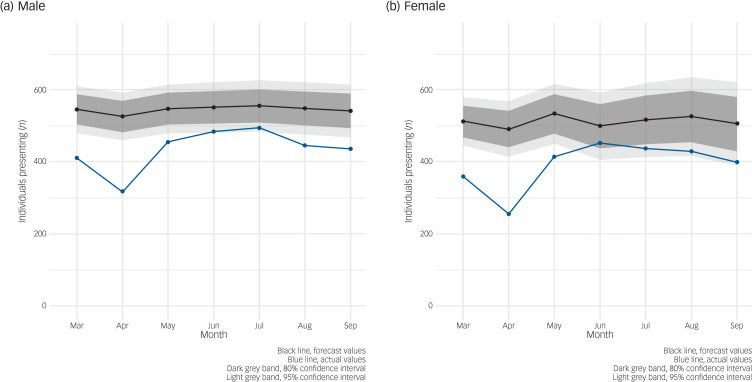
Auto-regressive integrated moving average (ARIMA) illustrating forecast versus actual numbers of individuals presenting with self-harm or ideation (thoughts of self-harm or suicide) by sex during the first seven months of COVID-19 pandemic/restrictions in Northern Ireland. (a) Male. (b) Female.

**Fig. 4:**
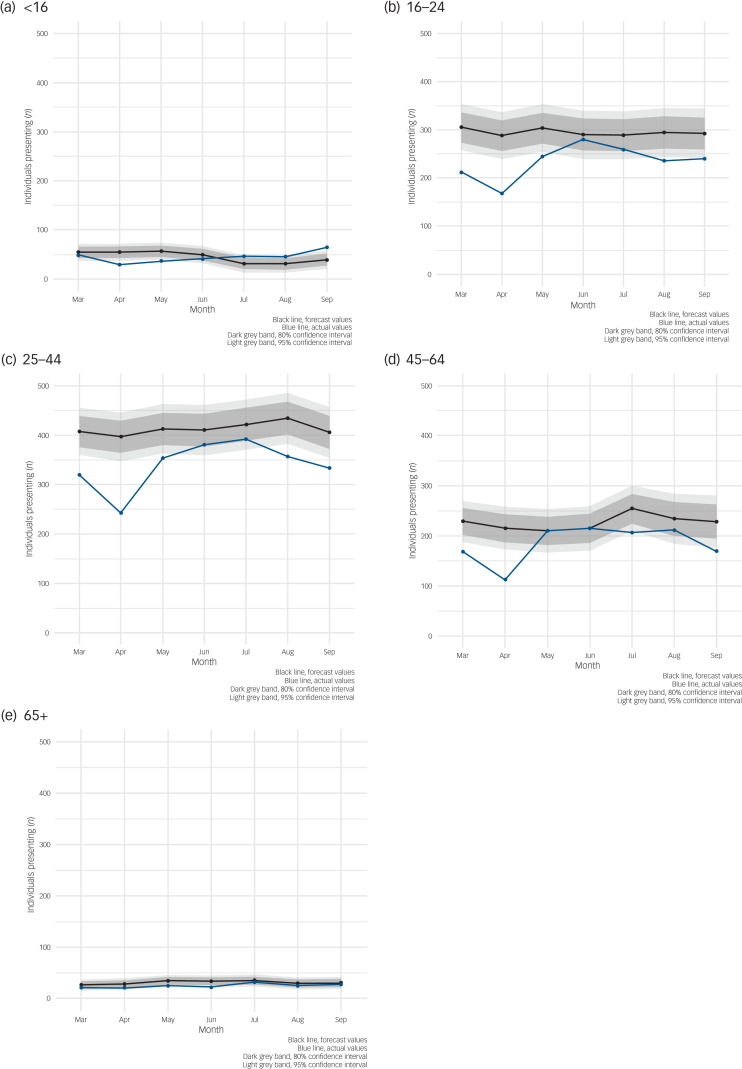
Auto-regressive integrated moving average (ARIMA) illustrating forecast versus actual numbers of individuals presenting with self-harm or ideation (thoughts of self-harm or suicide) during the first seven months of COVID-19 pandemic/restrictions in Northern Ireland stratified by age group. (a) <16 years. (b) 16–24 years. (c) 25–44 years. (d) 45–64 years. (e) 65+ years.

**Fig. 5:**
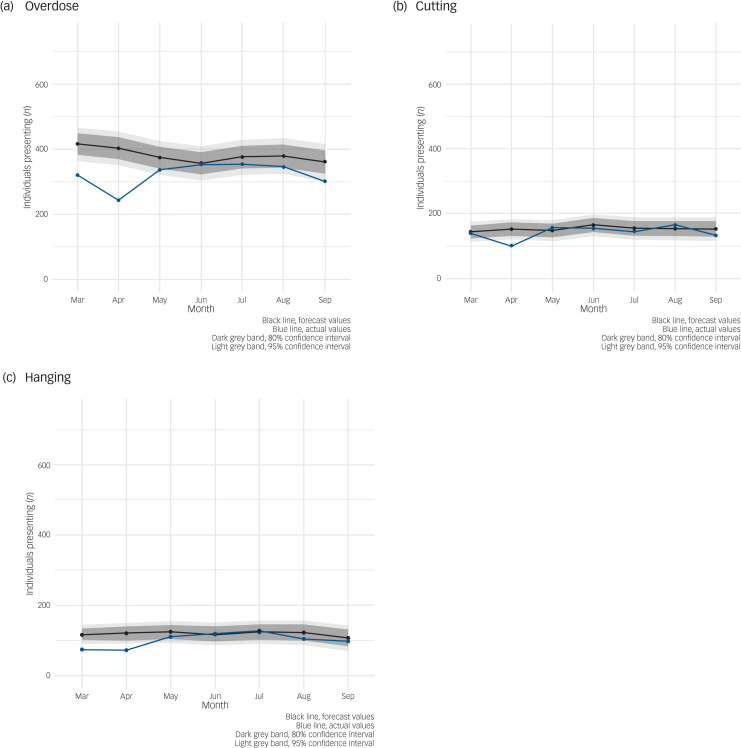
Auto-regressive integrated moving average (ARIMA) illustrating forecast versus actual numbers of individuals presenting with self-harm or ideation (thoughts of self-harm or suicide) during the first seven months of COVID-19 pandemic/restrictions in Northern Ireland stratified by method. (a) Overdose. (b) Cutting. (c) Hanging and other.

ARIMA models for trend were then trained by the range of demographic and presentation-related factors (Figs. 3–5, Supplementary Figs. 8–10). For these models, self-harm and ideation were grouped together because of the small numbers of outcomes in some subgroups. Figure 3 shows the expected versus the observed number of individuals presenting with self-harm or ideation from March to September 2020 stratified by sex. Males and females both exhibited a large drop in presentations in the first three months of the pandemic, with numbers of presenting females returning to expected levels from June 2020, whereas the number of presenting males continued to be slightly lower than expected up to September 2020.

When stratified by age most age groups followed the same pattern of a large drop in the first two or three months followed by a return to expected levels, except for those aged over 65 years, whose presentations for self-harm/ideation appeared to be unaffected by the pandemic as they continued to present within expected levels, and those aged under 16 years, where we see a much smaller drop in number of individuals presenting in March 2020 but a higher than expected number of individuals presenting by September 2020 (Fig. 4).

The substantial drop in presentations for self-harm or ideation in the first two or three months of the pandemic is not observed for individuals who live alone (Supplementary Fig. 8) or those who live in affluent areas (Supplementary Fig. 10), where the numbers remained as expected.

Figure 5 examines differences in trends in the number of individuals presenting, stratified by self-harm method. The biggest drop in the first two months is in those individuals presenting with ‘overdose’. The numbers of individuals presenting with ‘cutting’ or ‘hanging/other’ remained mainly as expected.

## Discussion

### Main findings

The number of individuals presenting with self-harm or ideation (i.e. thoughts of self-harm or suicide) had been increasing steadily for the 7 years pre-pandemic in Northern Ireland. Predictive modelling shows that the number of individuals presenting with self-harm or ideation fell during the first two to three months of the COVID-19 pandemic but was generally back within the expected range (as calculated from trends in the 7 years prior to the COVID-19 pandemic/restrictions) by June 2020. This is similar to data reported in a smaller study in England, which was limited in its comparison data, comparing numbers of presentations in 2019 with 2020 only.^8^ A drop in secondary care service utilisation for other conditions during the first few months of the COVID-19 pandemic/restrictions has been evidenced in a range of studies.^14–17^ However, a Welsh study examining both primary care and secondary care contacts for self-harm during the pandemic found primary care contacts with self-harm reduced disproportionately compared with non-self-harm contacts, suggesting a reduction in help-seeking behaviour within this cohort, which may be reflected in the decrease in presentations observed in this study.^7^

However, after stratification, there were differences observed in the demographic profile of those presenting with self-harm or ideation, with slightly fewer men than women presenting, presentations remaining unaffected in individuals aged over 65 years and those who live alone or in affluent areas and presentations appearing to increase in those aged under 16 years. Groups that saw little change had small numbers of presentations. The expected tsunami of mental ill health is not evidenced in an increase in hospital-presenting self-harm or ideation. It is not known whether these differences will persist into long-term effects of the pandemic/restrictions.

### Interpretation

In five out of the first seven months of the pandemic, male presentations to emergency departments with self-harm or ideation were lower than expected. This may be reflective of the increased ‘shielding’ among men, who are known to be at a higher risk of poor COVID-19 outcomes compared with women.^18^ A US study found that individuals who overestimated the risk of COVID-19 were more likely to practise household isolation.^19^ Another explanation may be that more men who previously would have presented with self-harm or ideation died on their first suicide attempt, although this does not appear to be reflected in national statistics on death by suicide.^20^ The full extent of the impact of the pandemic on suicide is not yet fully understood owing to delays in suicide reporting and coronial investigations.^4^ Lastly, the pandemic may have had a positive effect on men's mental health, at least during the initial seven months, where the collective nature of the stressor, coupled with increased time with family, and ‘furlough’ schemes that provided financial support for those who were unable to work, may have temporarily addressed the stressors associated with suicidal thoughts and supported good mental health.^21,22^

Age differences in the presentation profile of self-harm or ideation were also observed, with an increasing trend in the number of individuals presenting aged under 16 years. This supports reports from UK doctors at the time suggesting that more young people presented to emergency departments with self-harm.^23^ A recent systematic review has suggested that the COVID-19 pandemic has had a particularly negative effect on child and adolescent mental health, with increased anxiety, depression and loneliness.^24^ During the first seven months of the pandemic covered in this study, schools in Northern Ireland remained closed and children were educated using a mixture of home-schooling and distance learning, with schools and teachers utilising video call technology and remote learning applications. This separation from their peers and disruption to usual structure may have had a negative impact on their mental health. This may have become manifest in self-harm or ideation. In addition, children with additional needs, such as those with neuropsychiatric and neurodevelopmental disorders, may have found it difficult to cope with the initial pandemic/restrictions, which led to school closures, a reduction in usual care and reduced social care support, and this may have resulted in an increase in self-harm. The pandemic and associated restrictions have also been shown to have been particularly difficult for young people with existing mental health conditions.^25^ This cohort may require additional attention post-pandemic and this should be taken into consideration when planning mental health services.^26^ Differences by method of self-harm are not overwhelmingly significant, although the ARIMA models are suggestive of a larger decrease in presentations involving ‘overdose’. This is supported by the small English study, which suggests a larger decrease in presentations involving ‘self-poisoning’ compared with other methods.^8^

### Strengths and limitations

This study utilised eight years of data from the NIRSH, the world's only population-wide registry of self-harm and ideation, linked to individual-level health card registration data, to examine the impact of the COVID-19 pandemic and associated restrictions on hospital-presenting self-harm and self-harm/suicide ideation in Northern Ireland. The use of individual-level data allowed for an accurate analysis of numbers of individuals presenting to emergency departments across the country each month. The NIRSH uses standardised data collection practices and covers all emergency departments in Northern Ireland, providing a verified rich source information on presentations for self-harm and ideation at a population level. The information collected also facilitated the investigation of the impact of the pandemic/restrictions on subgroups of the population, and thus identification of populations at risk, such as males and children under 16 years, that could not be perceived from the full cohort analysis. Analysis was limited to the data included within the NIRSH, which is only those individuals who presented to an emergency department. Many individuals who self-harm do not present to secondary care. Therefore, the decline in presentations in the first few months of the pandemic is not necessarily reflective of a decrease in self-harm or ideation, but may be reflective of a decrease in service utilisation during that time. The use of longitudinal data and time series analysis (ARIMA) allowed for consideration of long-term trends and seasonality in self-harm behaviours. This design provides more reliable evidence of a change in expected patterns than simpler before-and-after comparisons, which may neglect to account for the pre-existing increase in many indicators for mental ill health.^8^

## Data Availability

The data used in this study cannot be publicly deposited. Project-specific data-sets are created and destroyed in line with the Administrative Data Research Centre Northern Ireland guidelines. Metadata for each project is available from the corresponding author on request. Access to the data-sets used can be obtained on application to the Health and Social Care Northern Ireland Business Service Organisation's Honest Broker Service (https://hscbusiness.hscni.net/services/2454.htm).

## Supplementary material

For supplementary material accompanying this paper visit https://doi.org/10.1192/bjp.2023.76. click here to view supplementary material
