# Supplementary material for: Impact of the COVID-19 pandemic on self-harm and self-harm/suicide ideation: population-wide data linkage study and time series analysis
Source: Br J Psychiatry. 2023 Nov;223(5):509–17. doi: 10.1192/bjp.2023.76 (PMC10895516; doi:10.1192/bjp.2023.76)
Supplement: Supplementary file 1 [file S0007125023000764sup001.docx]

Figure S1: Number of individuals presenting with self-harm or ideation stratified per 100,00 (using population mid-year estimates) 2012-2020 (dashed vertical line depicts onset of pandemic March 2020)

|  | **Individuals presenting/100k**  **(presentations/100k)** | | | | | | | | | | | | | | | | | |
| --- | --- | --- | --- | --- | --- | --- | --- | --- | --- | --- | --- | --- | --- | --- | --- | --- | --- | --- |
| **Month** | **2012** | | **2013** | | **2014** | | **2015** | | **2016** | | **2017** | | **2018** | | **2019** | | **2020** | |
| Jan | no data | | 43.74 | (54.58) | 47.26 | (59.42) | 44.54 | (55.79) | 51.31 | (64.29) | 48.84 | (60.69) | 52.75 | (67.39) | 56.38 | (72.15) | 56.16 | (70.48) |
| Feb | no data | | 42.13 | (49.96) | 39.12 | (49.00) | 45.59 | (57.17) | 50.74 | (63.95) | 50.03 | (63.25) | 47.72 | (62.41) | 47.77 | (59.90) | 58.11 | (71.84) |
| Mar | no data | | 44.54 | (55.38) | 47.15 | (60.51) | 47.53 | (58.89) | 52.82 | (66.76) | 50.03 | (64.17) | 55.93 | (70.80) | 60.13 | (76.66) | 45.56 | (56.45) |
| Apr | 39.06 | (47.54) | 44.27 | (54.04) | 48.18 | (61.61) | 47.59 | (57.12) | 46.64 | (59.51) | 52.14 | (67.36) | 51.02 | (62.70) | 58.08 | (72.73) | 33.97 | (44.20) |
| May | 40.22 | (50.28) | 42.45 | (52.38) | 45.89 | (57.68) | 48.53 | (60.05) | 51.59 | (67.26) | 50.95 | (65.93) | 58.31 | (73.00) | 61.60 | (77.77) | 51.48 | (65.15) |
| Jun | 40.12 | (49.91) | 44.49 | (54.69) | 44.64 | (55.99) | 48.75 | (62.16) | 48.89 | (63.50) | 49.29 | (60.86) | 56.80 | (72.01) | 61.19 | (79.35) | 55.50 | (70.18) |
| Jul | 42.17 | (52.38) | 49.16 | (60.86) | 44.91 | (57.90) | 45.54 | (58.83) | 47.54 | (60.13) | 53.74 | (66.50) | 58.65 | (75.89) | 59.31 | (77.36) | 55.39 | (69.35) |
| Aug | 39.54 | (49.54) | 44.97 | (55.17) | 46.60 | (60.51) | 45.04 | (58.45) | 49.11 | (63.11) | 53.57 | (69.92) | 58.94 | (76.35) | 59.72 | (80.35) | 51.84 | (66.75) |
| Sep | 39.70 | (50.17) | 39.77 | (50.18) | 44.47 | (56.37) | 45.70 | (58.45) | 46.53 | (58.72) | 49.41 | (62.40) | 56.97 | (71.61) | 60.72 | (76.01) | 49.47 | (62.90) |
| Oct | 42.12 | (51.49) | 44.11 | (55.28) | 45.89 | (56.91) | 50.19 | (59.89) | 47.48 | (60.75) | 57.33 | (73.85) | 54.55 | (72.59) | 54.09 | (68.10) | no data | |
| Nov | 40.59 | (50.07) | 41.05 | (50.45) | 46.49 | (58.71) | 45.98 | (56.01) | 43.49 | (54.00) | 50.20 | (65.65) | 54.89 | (69.64) | 52.92 | (67.93) | no data | |
| Dec | 36.22 | (46.01) | 38.26 | (48.94) | 42.89 | (53.91) | 46.76 | (58.67) | 46.53 | (57.77) | 48.95 | (62.40) | 53.39 | (67.04) | 48.47 | (61.01) | no data | |

Table S1: Number of individuals presenting with self-harm or ideation stratified per 100,00 (using population mid-year estimates) each month from 2012-2020 (dashed vertical line depicts onset of pandemic March 2020)

Figure S2: Number of individuals presenting with self-harm or ideation stratified by gender 2012-2020 (dashed vertical line depicts onset of pandemic March 2020)


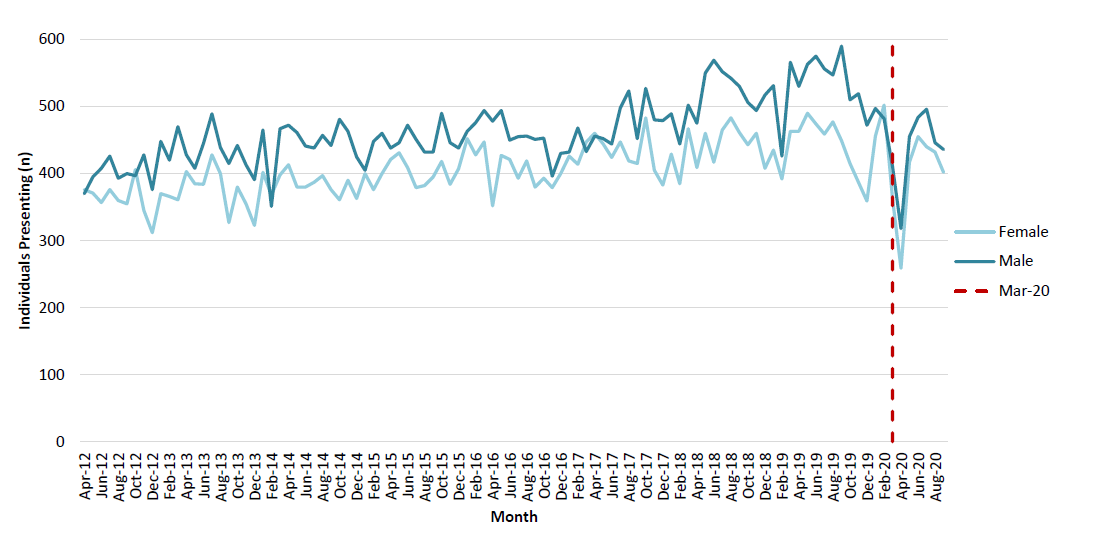


Figure S3: Number of individuals presenting with self-harm or ideation stratified by age group 2012-2020 (dashed vertical line depicts onset of pandemic March 2020)


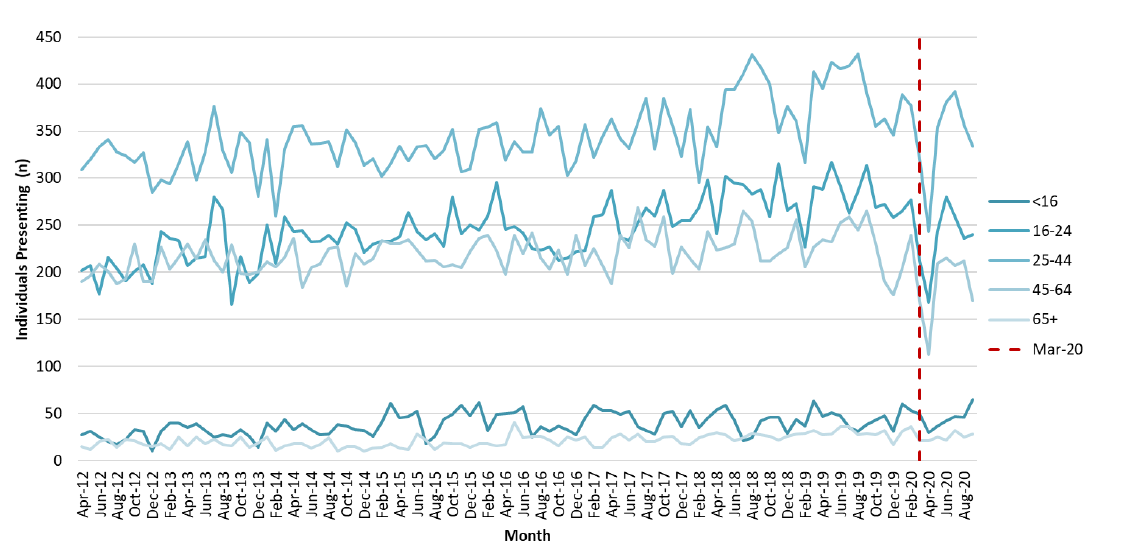


Figure S4: Number of individuals presenting with self-harm or ideation stratified by area deprivation 2012-2020 (dashed vertical line depicts onset of pandemic March 2020)


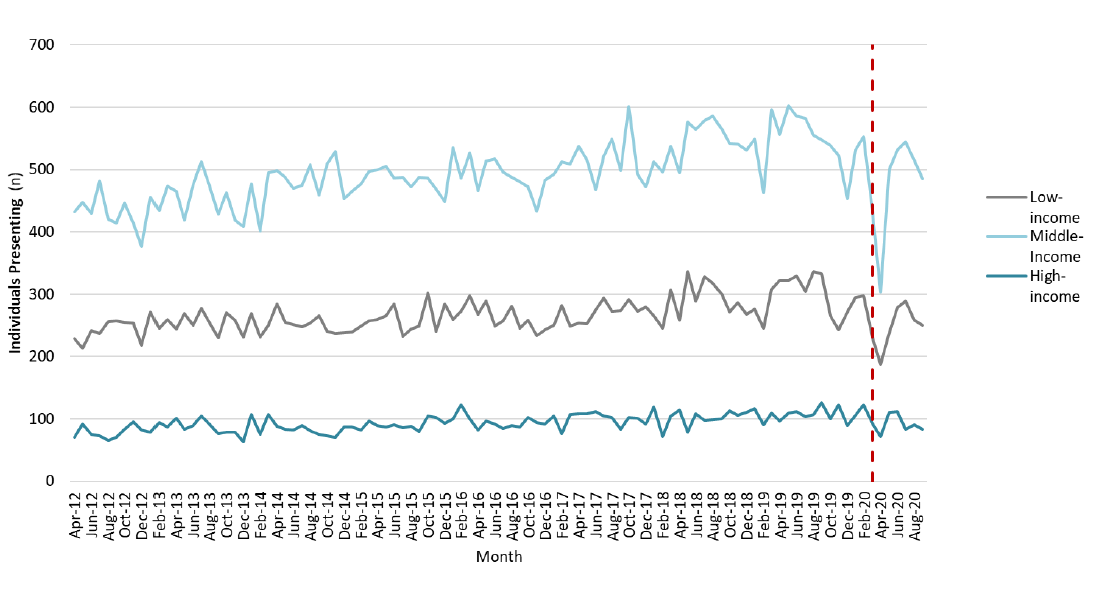


Figure S5: Number of individuals presenting with self-harm or ideation stratified by urban/rural 2012-2020 (dashed vertical line depicts onset of pandemic March 2020)


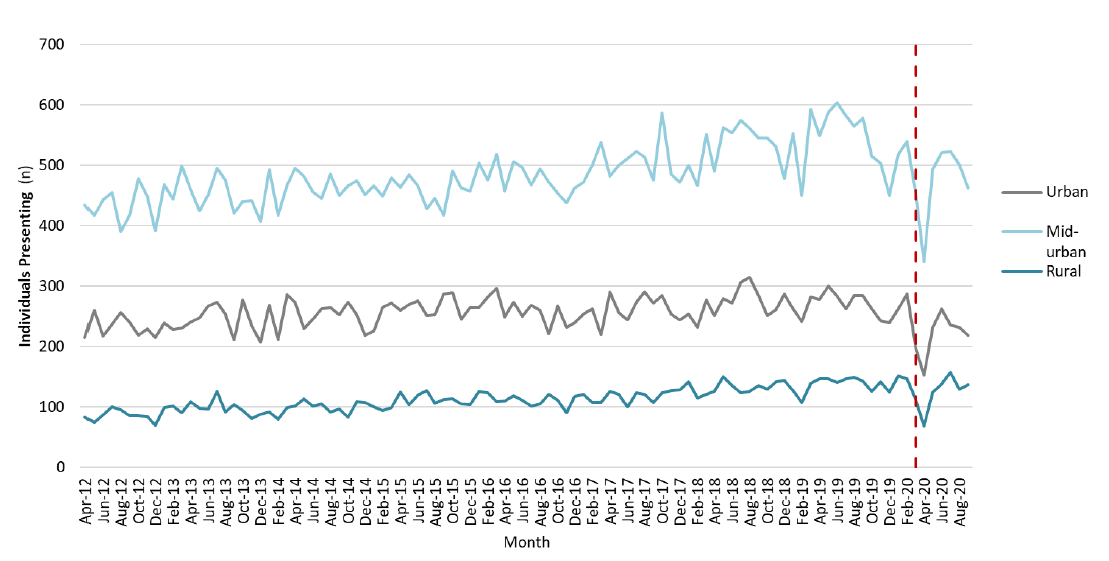


Figure S6: Number of individuals presenting with self-harm or ideation stratified by method 2012-2020 (dashed vertical line depicts onset of pandemic March 2020)


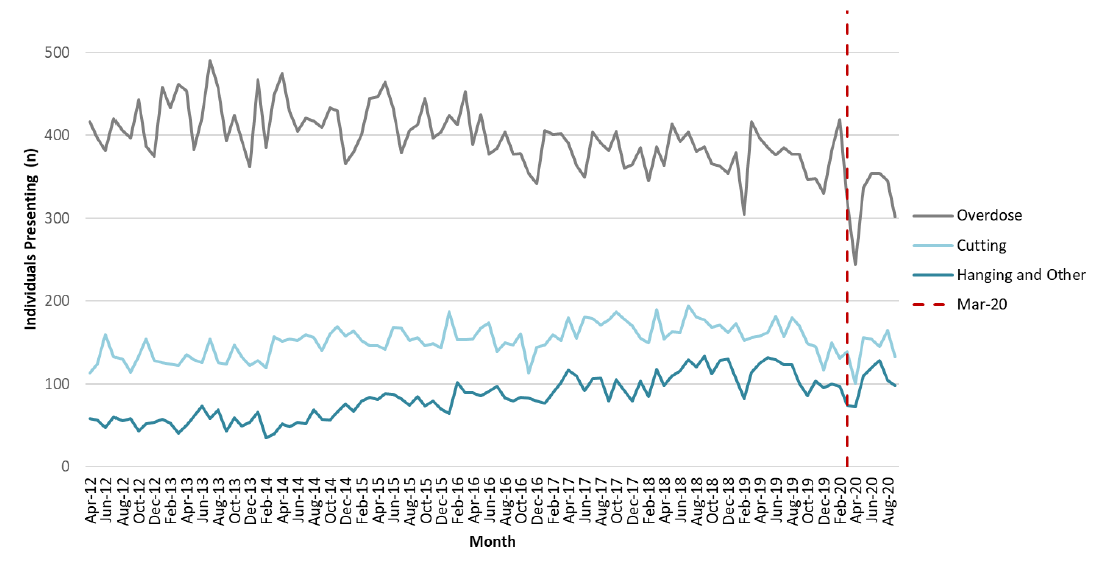


Figure S7: Number of individuals presenting with self-harm or ideation stratified by Household Occupancy 2012-2020 (dashed vertical line depicts onset of pandemic March 2020)


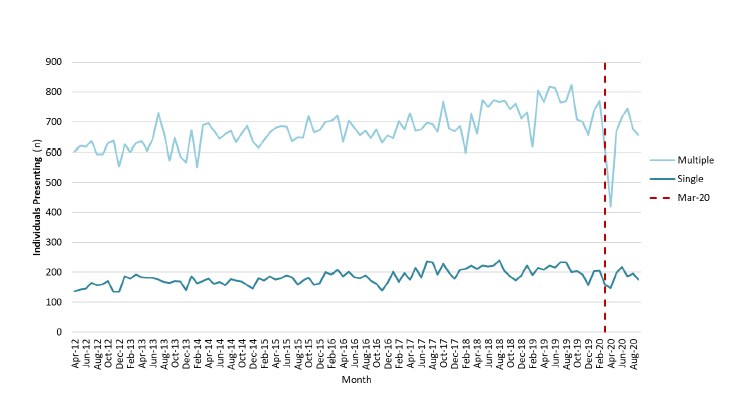


Figure S8: Auto regressive integrated moving average (ARIMA) illustrating forecast versus actual numbers of individuals presenting with self-harm or ideation during the first 7 months of COVID-19 pandemic/restrictions in Northern Ireland stratified by Household Occupancy: (a) single occupancy, (b) multiple occupancy (black line = forecast values, red line = observed values)

| 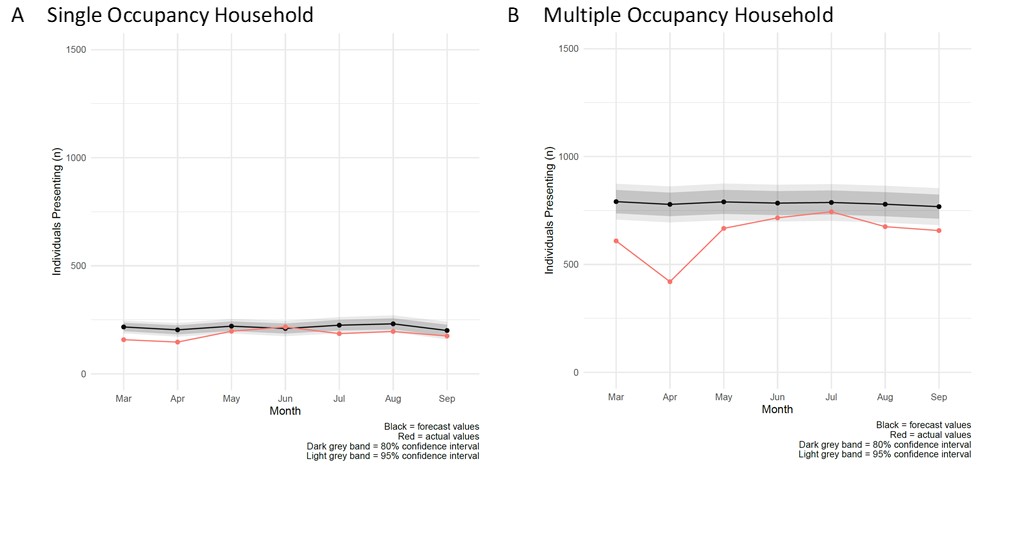 |  |  |  |
| --- | --- | --- | --- |
|  |  |  |  |
|  |  |  |  |
|  |  |  |  |

Figure S9: Auto regressive integrated moving average (ARIMA) illustrating forecast versus actual numbers of individuals presenting with self-harm or ideation during the first 7 months of COVID-19 pandemic/restrictions in Northern Ireland stratified by urbanicity: (a) Rural, (b) Mid-urban, (c) Urban (black line = forecast values, red line = observed values)

| A | Rural | B | Mid-urban |
| --- | --- | --- | --- |
|  | 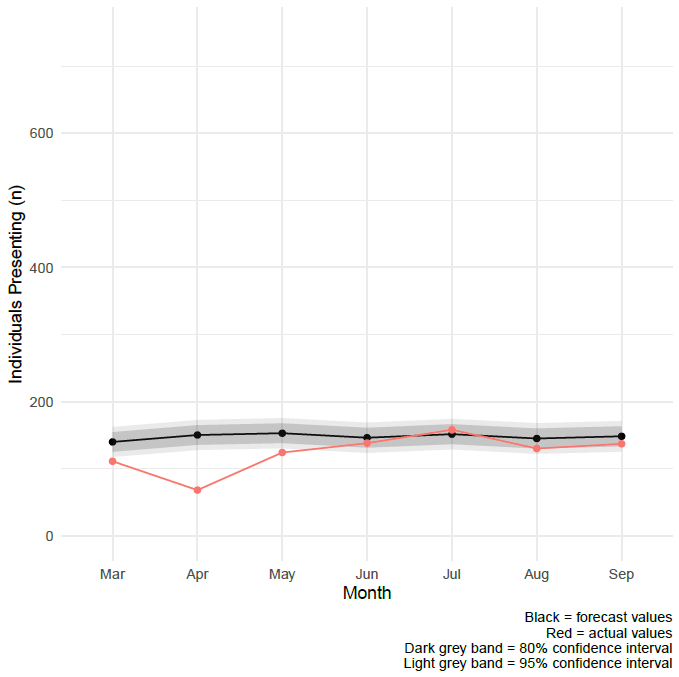 |  | 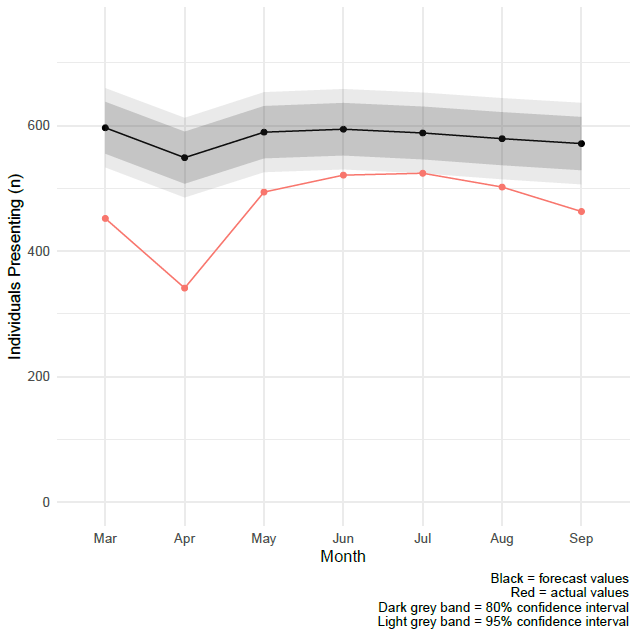 |
| C | Urban |  |  |
|  | 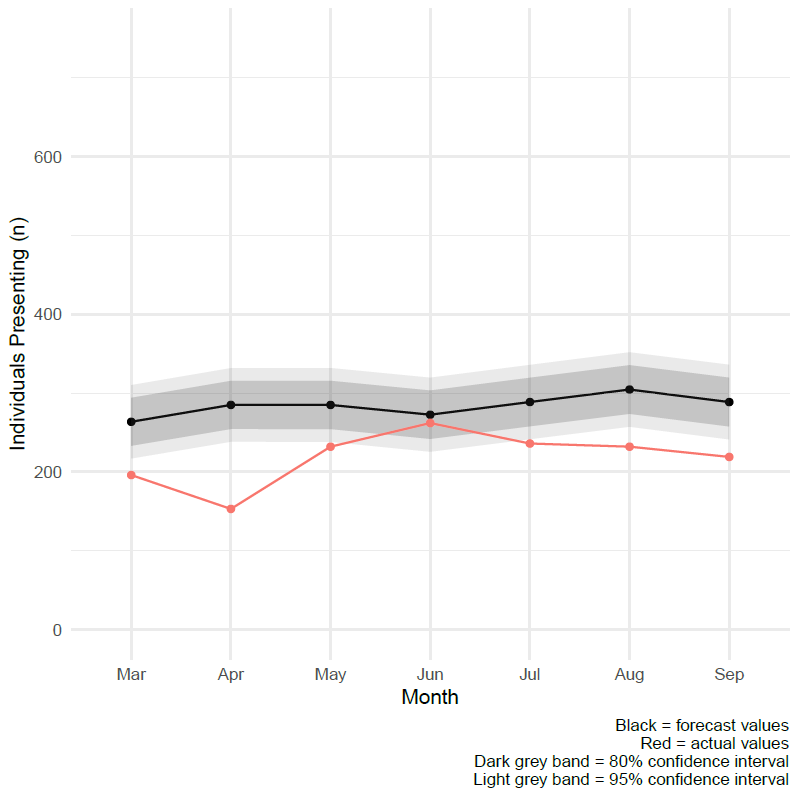 |  |  |

Figure S10: Auto regressive integrated moving average (ARIMA) illustrating forecast versus actual numbers of individuals presenting with self-harm or ideation during the first 7 months of COVID-19 pandemic/restrictions in Northern Ireland stratified by area level income Deprivation: (a) low income, (b) middle income, (c) high income (black line = forecast values, red line = observed values)

| 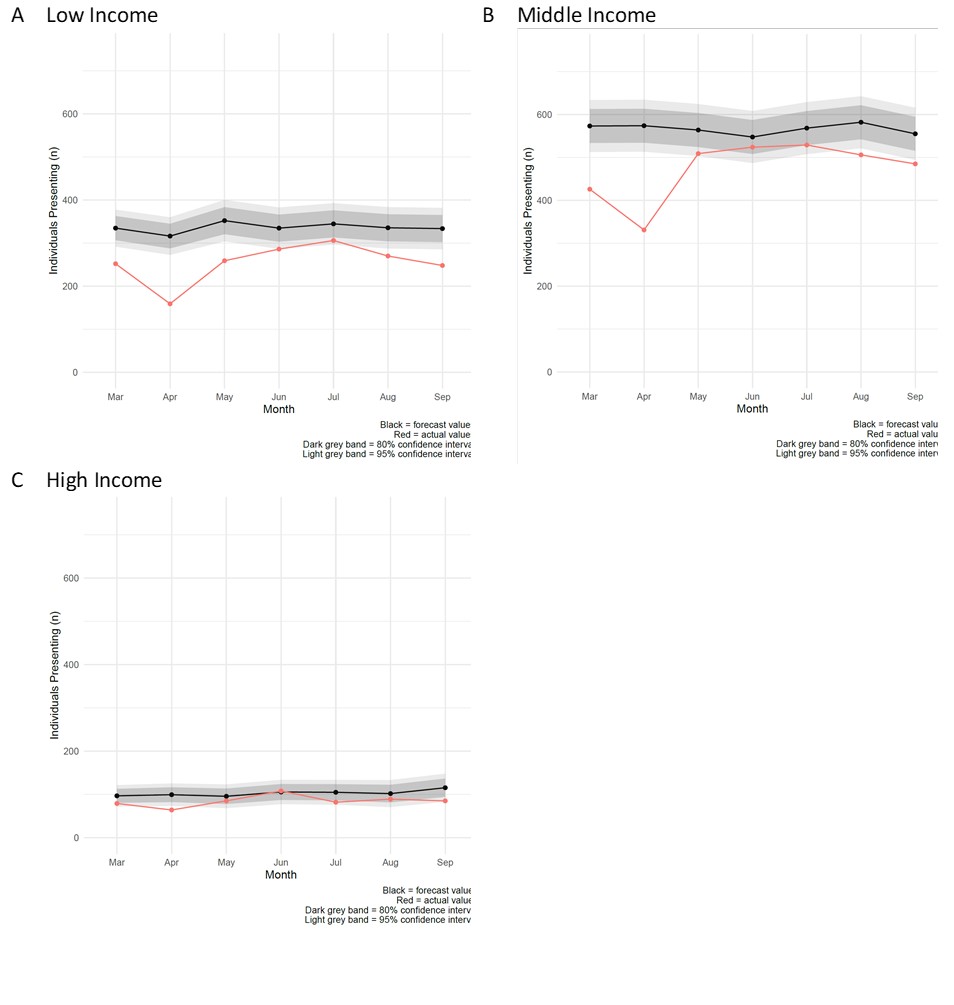 |  |  |  |
| --- | --- | --- | --- |
|  |  |  |  |
